# Non-volatile resistive switching mechanism in single-layer MoS_2_ memristors: insights from *ab initio* modelling of Au and MoS_2_ interfaces†

**DOI:** 10.1039/d3na00045a

**Published:** 2023-07-21

**Authors:** Gabriele Boschetto, Stefania Carapezzi, Aida Todri-Sanial

**Affiliations:** a Laboratory of Computer Science, Robotics, and Microelectronics, University of Montpellier, CNRS 161 Rue Ada 34095 Montpellier France gabriele.boschetto@lirmm.fr aida.todri@lirmm.fr; b Department of Electrical Engineering, Eindhoven University of Technology Groene Loper 3 5612 AE Eindhoven Netherlands

## Abstract

Non-volatile memristive devices based on two-dimensional (2D) layered materials provide an attractive alternative to conventional flash memory chips. Single-layer semiconductors, such as monolayer molybdenum disulphide (ML-MoS_2_), enable the aggressive downscaling of devices towards greater system integration density. The “atomristor”, the most compact device to date, has been shown to undergo a resistive switching between its high-resistance (HRS) and low-resistance (LRS) states of several orders of magnitude. The main hypothesis behind its working mechanism relies on the migration of sulphur vacancies in the proximity of the metal contact during device operation, thus inducing the variation of the Schottky barrier at the metal–semiconductor interface. However, the interface physics is not yet fully understood: other hypotheses were proposed, involving the migration of metal atoms from the electrode. In this work, we aim to elucidate the mechanism of the resistive switching in the atomristor. We carry out density functional theory (DFT) simulations on model Au and ML-MoS_2_ interfaces with and without the presence of point defects, either vacancies or substitutions. To construct realistic interfaces, we combine DFT with Green's function surface simulations. Our findings reveal that it is not the mere presence of S vacancies but rather the migration of Au atoms from the electrode to MoS_2_ that modulate the interface barrier. Indeed, Au atoms act as conductive “bridges”, thus facilitating the flow of charge between the two materials.

## Abstract 2

The migration of Au atoms to fill the S vacancies in MoS_2_ memristors increases the electron injection rate and reduces the contact resistance at the metal–semiconductor interface, thus causing the resistive switch.

## Graphical abstract

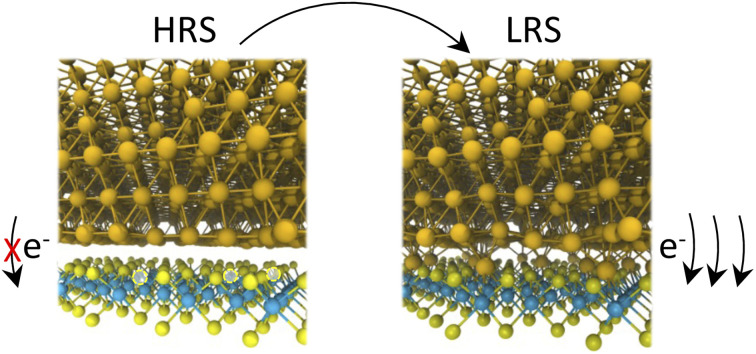

## 1 Introduction

Memristors are emerging nanoelectronic devices whose non-volatile properties could enable super-fast and ultra-low power memory chips.^1,2^ Additionally, memristive devices could also be employed for data encryption^3,4^ and as synaptic units in the context of brain-inspired (neuromorphic) computing technologies.^5–8^

Memory devices beyond the conventional silicon flash storage, such as resistive random access memories (RRAMs), are typically composed of a core material (*i.e.*, a metal oxide) sandwiched between two metal electrodes.^9,10^ At the device level, the modulation of the electrical resistance (resistive switching) is achieved by applying external electric stimuli. At the material level, the working mechanism of RRAMs involves the formation and rupture of conductive filaments of either internal ions or ionic vacancies in the oxide channel, mediated by the electric field.^11,12^ Such a conductive filament is then able to bridge (decouple) the top and bottom electrodes, thus allowing (hindering) electrical charge to flow through the device.

Recently, layered two-dimensional (2D) materials have also been used as novel core materials to develop and fabricate memory devices.^7,8,13,14^ For instance, non-volatile resistive switching has been observed in devices based on graphene and derivatives,^15,16^ and on transition metal dichalcogenides (TMDs), such as molybdenum disulphide (MoS_2_). Similarly to graphene, MoS_2_ can be thinned down to a single layer, which shows several desirable mechanical, electrical, and optical properties, thus making it the ideal candidate to be used in a wide range of (opto)electronic devices.^17–21^ Surprisingly, recent work revealed that vertical 2D memristors could also work by employing only one single layer of MoS_2_ sandwiched between two Au electrodes.^22,23^ Such a compact device, also known as “atomristor”, has been shown to function at low voltages (≤1 V), thus having the great advantage of being low-power while enabling denser system integration. Moreover, the device retained its properties even after several bending cycles, making it suitable to be used in flexible electronics.

Although other examples of memristors based on single (or few) layer(s) of MoS_2_ have been reported, the mechanism of the resistive switching in these devices is still not well understood. In general, several factors are thought to come into play, such as the device architecture, the nature of the metal electrodes, whether MoS_2_ is employed as multi-layer, single-layer or few-layer, its crystallinity, and if the material has been somehow functionalized.^24^ For instance, the formation of a conductive filament has been hypothesized in a recent memtransistor device based on multilayer MoS_2_ and with Ag electrodes,^25^ as well as in another vertical device based on bilayer MoS_2_ sandwiched between Cu and Au electrodes.^26^ In both cases, the conductive filament has been attributed to ions migrating from the Ag and Cu active electrodes. Sangwan *et al.*^27^ reported a lateral two-terminal device in which the resistive switching was mediated by the presence and orientation of grain boundaries in monolayer (ML) MoS_2_. Similarly, Wang *et al.*^28^ reported a top-gated memory device with polycrystalline MoS_2_ as the channel, in which the proposed working mechanism involves the migration of sulphur defects (mainly di-vacancies) across the grain boundaries. S vacancies often occur in MoS_2_ samples obtained by chemical vapour deposition (CVD) and following annealing, and can reach very high densities: typically, on the order of 10^13^ cm^−2^.^29^ With such a high defect density, it is clear that the material is far from being pristine.

It appears that, regardless of the device architecture, S vacancies inevitably occurring in MoS_2_ are thought to be crucial to trigger the non-volatile resistive switching. Indeed, the main hypothesis behind the working mechanism of the atomristor involves the migration of S vacancies, possibly mediated by the electric field and/or temperature, which diffuse perpendicularly to the electrodes forming filament-like nano-conductive links.^22,30,31^ Then, the conductive links modulate the Schottky barrier at the electrode interface. Indeed, an impressive switch of several orders of magnitude was observed from the initial high-resistance state (HRS) to the low-resistance state (LRS). Moreover, devices based on ML-MoS_2_ with multilevel resistive switching have also been reported.^30^ It is important to point out that such a mechanism is rather unique, and it is different from that occurring in oxide-based devices. Recently, the hypothesis behind the working mechanism of the atomristor has been challenged by experimental studies suggesting that such a significant variation of the electrical resistance is not due to S vacancies alone, but rather to Au atoms migrating from the metal electrode and filling the S vacancies.^32,33^ This is corroborated by the fact that Au electrodes are known to easily feature adatoms on the surface.^34^

This paper aims to contribute towards the better understanding of the non-volatile resistive switching mechanism in memristor devices based on ML-MoS_2_. Given the hypotheses currently at study, both involving migration and diffusion of atoms at the interface between MoS_2_ and the Au electrode, we carry out atomistic computer simulations within the framework of density functional theory (DFT). Such a simulation approach has been already used to study similar systems and provided invaluable insights into the chemistry and physics of interfaces.^35–39^ Here, we construct realistic Au/ML-MoS_2_ model interfaces and we investigate how the presence of defects and Au adatoms at the materials' interface impact the complex physics of memristors. To realistically simulate semi-infinite metal electrodes rather than metal slabs, we employ the Green's function surface model approach. We successfully used this simulation framework in a previous work on defective ML-MoS_2_ interfaces.^39^ With our combined DFT-Green's function surface simulations, we aim to provide the necessary theoretical justification to the hypotheses of the non-volatile resistive switching mechanism in single-layer MoS_2_ memristors.

This paper is organized as follows: in the following section (Simulations details), we provide an overview of our theoretical approach and simulation setup. Then, in Results and discussion, we present the results of our simulations with a discussion in the context of the atomristor. The final section contains the Conclusions.

## 2 Simulation details

In this work, we focused on defective ML-MoS_2_ either with point defects or Au adatoms in top contact with the Au(111) electrode. First, we looked at the ideal defect-free interface, in which we initially introduced one single point defect (either one S vacancy or one Au atom filling such vacancy). Then, we systematically increased the concentration of such defects and we varied their position to assess the effect of clustering. From this point onward, unless otherwise specified, we will simply refer to MoS_2_ to indicate a single-layer structure. Also, we will refer to each Au/MoS_2_ interface with the name of the defect introduced therein: for instance, V_S_ and Au_S_ correspond to interfaces with one S vacancy and one Au atom filling such a vacancy, respectively. To increase the concentration of defects, we doubled and multiplied by four the number of both S vacancies and Au atoms per unit cell: 2 × V_S_ and 2 × Au_S_, and 4 × V_S_ and 4 × Au_S_. We point out that here defects were randomly introduced without any clustering. However, we also investigated the effect of defect clustering, and we considered S di-vacancies either in-plane (V^P^_S2_) or axial (V^A^_S2_). Equally, we considered axial and in-plane clusters of Au atoms: Au2^A^_S2_ and Au2^P^_S2_, respectively. Finally, we considered a large 4-atom cluster of S vacancies (V_S4_).

**Fig. 1:**
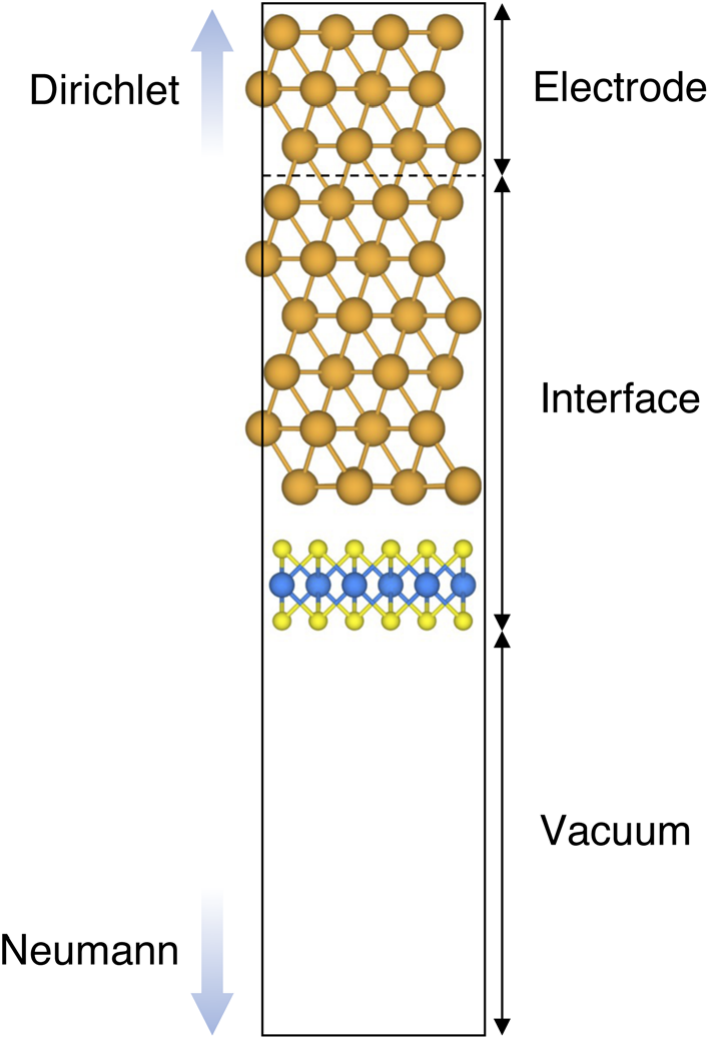
Representative example of the Au/MoS_2_ interface, modelled with the Green's function surface approach. The system can be divided into three parts: (1) the electrode (to which Dirichlet boundary conditions were applied), (2) the central part, consisting of 6 atomic layers of Au coupled with single-layer MoS_2_, and (3) the vacuum region, to which Neumann boundary conditions were applied.

We constructed Au/MoS_2_ interfaces by matching the crystal lattice of Au(111) to that of MoS_2_, which led to a small unavoidable mean absolute strain of ∼3% in the Au electrode. The structure of MoS_2_ was kept free of any strain and with a calculated in-plane lattice constant of 3.16 Å, which matches very well with experimental data.^40^ It is worth noting that strain on the Au electrode may vary the local registry between Au and MoS_2_ lattices,^36^ and consequently may also affect the computed average interface distances. Nevertheless, as we will discuss in the following section, our results compare well with experimental data^22^ and are well within the range of values found in other computational studies in the literature.^36,37,41^ To construct such interfaces, we considered a 3 × 3 orthorhombic MoS_2_ supercell, thus allowing us to keep the concentration of single point defects in the experimental range of ∼10^13^ cm^−2^ (equal to a defect density of 2.7%). Such a concentration corresponds to having one single point defect per unit cell, whereas when having two and four the defect density becomes 5.5 and 11%, respectively. The orthorhombic simulation cells of the interfaces had the following dimensions: *a* = 16.4219 Å, *b* = 9.4812 Å, and *c* = 45.5679 Å. A representative interface structure is shown in Fig. 1. Finally, we point out that effects arising from the Moiré pattern between Au(111) and MoS_2_, as well as the herringbone reconstruction of Au, were not taken into consideration in our simulations.

Atomistic simulations were carried out in the framework of density functional theory (DFT) with QuantumATK atomic-scale modelling software.^42,43^ All our spin-polarized calculations were performed in vacuum using the Perdew–Burke–Ernzerhof (PBE) exchange-correlation functional,^44^ and to model core electrons we used norm-conserving pseudopotentials from the PseudoDojo library.^45^ The linear combination of atomic orbitals (LCAO) approach was used throughout the work. Upon validation of the simulation settings,^39^ we chose a density-mesh cut-off of 150 Ry and the QuantumATK-optimized Medium basis set. A *k*-point mesh defined by a 2 × 3 Monkhorst–Pack (MP) grid^46^ was used to carry out geometry optimizations, which we increased up to 8 × 12 when computing device density of states (DOS). Geometry optimizations were converged with a maximum allowed atomic force threshold of 0.05 eV Å^−1^. To take into account long-range dispersion interactions, all the simulations were carried out by including the Grimme's D2 dispersion correction.^47^ Au/MoS_2_ interfaces were modelled *via* the Green's function surface approach as implemented in QuantumATK, which allowed us to construct semi-infinite (fully periodic in one direction) Au surfaces rather than rely on metal slabs.^48^ To carry out such simulations, we considered six atomic layers of Au coupled with MoS_2_, to which we imposed Dirichlet boundary conditions in the direction of the bulk Au electrode and Neumann boundary conditions in the vacuum direction (see Fig. 1). A total of 138 *k*-points along the direction of the Au electrode were chosen.

Adhesion energies (*E*_ad_) of MoS_2_ (pristine and defective) on the Au electrode were computed as:

1 *E*_ad_ = *E*_Au/MoS_2__ − (*E*_MoS_2__ + *E*_Au_)

where *E*_Au/MoS_2__ is the total energy of the Au/MoS_2_ interface, whereas *E*_MoS_2__ and *E*_Au_ are the total energies of the isolated MoS_2_ and Au fragments, respectively. As we carried out our simulations by using the LCAO approach with localized orbitals, we took into account the basis set superposition error (BSSE) by including the counterpoise correction^49^ in our total energy calculations.

## 3 Results and discussion

Here, we discuss on the quality of Au/MoS_2_ contacts by looking at their optimized interface structures, by computing figures of merit to assess their stability, and by predicting their tunnelling probabilities of electrons.

### 3.1 Structures and stability of defective interfaces

**Fig. 2:**
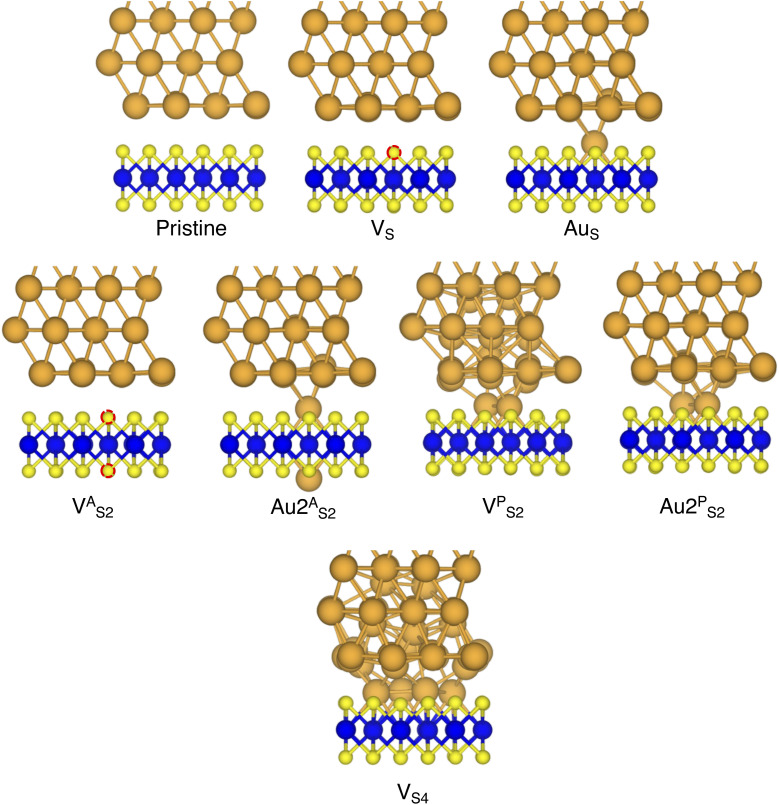
Optimized geometries with DFT of selected Au/MoS_2_ interfaces considered in this work. The pictures were taken in the vicinity of the contact area between the two materials. Interfaces V^P^_S2_ and V_S4_ refer to MoS_2_ with clusters of two and four S vacancies, although following geometry optimization such clusters were fully filled by Au atoms migrating from the above electrode.

In this part of the work, we focus on the stability of defective interfaces with respect to the pristine case by looking ad their DFT-optimized geometries and by computing adhesion energies (per surface Au atom). The atomic structure of selected interfaces are shown in Fig. 2, whereas average interface distances (*d*_Au–MoS_2__) and adhesion energies are presented in Table 1. For each interface, we decomposed the total *E*_ad_ into its dispersion (*E*^disp^_ad_) and electrostatic (*E*^elec^_ad_) contributions.

**Table 1:** Average interface distance (*d*_Au–MoS_2__) and adhesion energies (*E*_ad_) of the interfaces considered in this work. Adhesion energies were decomposed into dispersion and electrostatic contributions (*E*^disp^_ad_ and *E*^elec^_ad_, respectively), and were normalized per surface Au atom

Interface	*d*_Au–MoS_2__ (Å)	*E*_ad_ (eV)	*E* ^disp^_ad_ (eV)	*E* ^elec^_ad_ (eV)
Pristine^39^	2.71	−0.34	−0.39	+0.05
V_S_^39^	2.73	−0.34	−0.38	+0.04
Au_S_	2.72	−0.42	−0.41	−0.01
2 × V_S_^39^	2.70	−0.34	−0.37	+0.03
4 × V_S_^39^	2.67	−0.34	−0.36	+0.02
2 × Au_S_	2.74	−0.49	−0.43	−0.06
4 × Au_S_	2.76	−0.64	−0.45	−0.19
V^A^_S2_ (ref. 39)	2.70	−0.34	−0.38	+0.04
V^P^_S2_	—	−0.51	−0.40	−0.11
V_S4_	—	−0.68	−0.41	−0.27
Au2^A^_S2_	2.72	−0.42	−0.41	+0.01
Au2^P^_S2_	2.74	−0.49	−0.42	−0.07

**Fig. 3:**
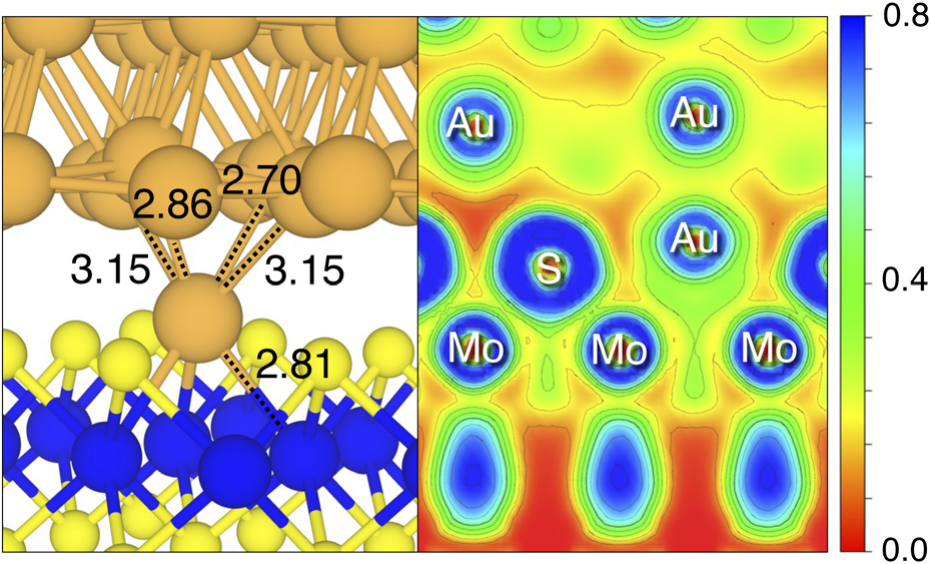
Optimized geometry of the Au_S_ interface (left image), in the proximity of the contact area. Relevant Au–Mo and Au–Au bond lengths (in angstrom) are shown. Contour plot of the ELF for the Au_S_ interface (right image) in the proximity of the contact area. The color scheme is RGB, and red corresponds to ELF = 0, whereas blue to ELF = 0.8.

**Fig. 4:**
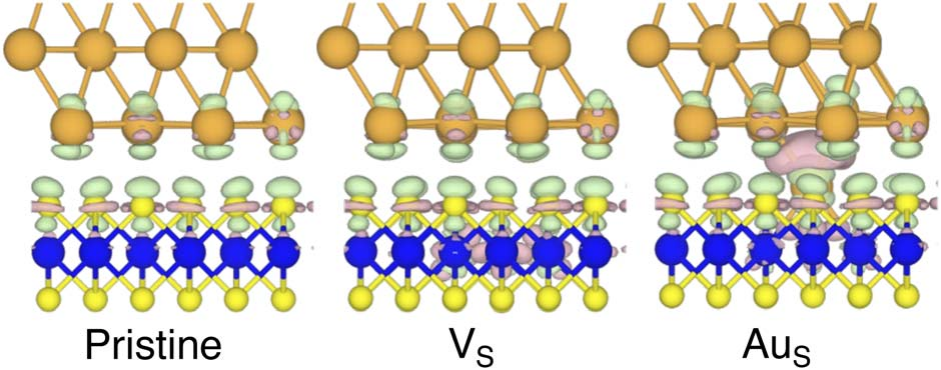
Electron density difference (EDD) maps of the pristine, V_S_, and Au_S_ interfaces in the vicinity of the contact area. Green colour corresponds to electron loss, whereas mauve colour to electron enrichment. The isovalue was set to 0.002 eV Å^−3^.

By looking at the optimized structure of the ideal defect-free interface, it is evident that there exist no chemical bonds between Au and MoS_2_: with an average *d*_Au–MoS_2__ of 2.71 Å, the interaction between Au and defect-free MoS_2_ is mainly driven by dispersion forces (van der Waals gap). This is corroborated by the *E*_ad_ of the interface, whose major contribution is given by *E*^disp^_ad_ with a small electrostatic repulsion. The weak interaction between Au and pristine MoS_2_ has been widely reported in the literature with theoretical simulations^36,41,50^ and in our previous work.^39^ However, experimental CVD-MoS_2_ samples are far from being pristine, and are mainly characterized by the presence of S vacancies. Our simulations show that such a defect at 2.7% density (approximately as in experimental samples^29^) does not significantly change the weak Au/MoS_2_ interaction: *d*_Au–MoS_2__ (2.73 Å) is comparable with that of the pristine interface, and the adhesion energy is still mainly given by weak dispersion forces. It is worth noting that the vdW gap between Au and CVD-MoS_2_ is also observed experimentally by looking at atomic resolution TEM images.^22,51^ Moreover, the size of the vdW gap that can be extracted by TEM images compares reasonably well with our computed value, thus further confirming our results. We then filled the S vacancy by introducing one Au atom and we optimized the full interface. By doing so, we considered the extra Au atom to be originally adsorbed on the metal electrode (extra adatom). Our simulations revealed that the bond length between Au and the Mo atoms of MoS_2_ is much longer than that between S and Mo (see Fig. 3) in the same material. We found the Au–Mo bond length to be 2.81 Å, which is 0.40 Å longer than the typical Mo–S bond length (2.41 Å). Thus, the Au atom sticks out of the basal plane of MoS_2_, allowing it to simultaneously bond also with the Au electrode. We found the bond lengths between the extra Au atom in MoS_2_ and the four closest Au atoms of the metal electrode to range between 3.15 and 2.70 Å (see Fig. 3). Interestingly, we point out that the Au–Au bond length in the electrode is 2.73 Å. The formation of a chemical bond between the extra Au atom and the metal electrode is further corroborated by the electron localization function (ELF), which we plotted in Fig. 3, and by the electron density difference (EDD) maps in Fig. 4. From the ELF plot, it is evident that there is a non-zero probability to locate electrons between the extra Au and the atoms of the electrode, whereas no direct chemical bonds are observed between S atoms and the metal electrode. Furthermore, EDD maps show a significant charge redistribution on the extra Au atom, whereas with pristine MoS_2_ and V_S_ only a limited charge redistribution is found at the materials' interface. Overall, the average *d*_Au–MoS_2__ in Au_S_ is comparable to that of the defect-free interface, although we point out that we observed a small rearrangement of the Au atoms of the electrode due to the presence of the extra Au atom above the basal plane of MoS_2_. Finally, the stronger interaction between MoS_2_ and the metal electrode is also corroborated by the stronger *E*_ad_, in which, in addition to the dispersion contribution, we also found a small electrostatic attraction (see Table 1). In conclusion, our simulations suggest that the extra Au atom that fills the S vacancy is able to act as a physical “bridge” between MoS_2_ and the metal electrode.

Then, we increased the concentration of both S vacancies and Au substitutions in MoS_2_. To do so, we doubled and multiplied by four the number of these defects in the simulation cell, resulting in a defect density of 5.5 and 11%, respectively. To avoid artificial clustering, we introduced defects randomly. Once again, we found a limited interaction between the metal electrode and MoS_2_ in both 2 × V_S_ and 4 × V_S_, suggesting that even at much higher concentrations the interface is characterized by a vdW gap. However, we observed a small (but negligible) decrease of *d*_Au–MoS_2__, from 2.71 Å (pristine interface) to 2.70 Å and 2.67 Å for 2 × V_S_ and 4 × V_S_, respectively. The weak interaction is also confirmed by *E*_ad_, whose value did not vary with respect to the pristine interface even at 11% density of S vacancies. On the contrary, we found that increasing the concentration of substitutional Au atoms leads to a significant increase in the electrode/MoS_2_ interaction. This is evident by looking at *E*_ad_, which increased from −0.42 eV in Au_S_ to −0.49 eV and −0.64 eV in 2 × Au_S_ and 4 × Au_S_, respectively. Interestingly, we point out that the *E*_ad_ in 4 × Au_S_ is essentially two times that in 4 × V_S_. It is also worth noting that the electrostatic attraction contribution in the adhesion energy increases with respect to the concentration of substitutional Au atoms: at the highest Au defect density, it accounts for roughly 30% of the total *E*_ad_. This is further proof of the capability of substitutional Au atoms to couple the metal electrode to MoS_2_ by forming strong chemical bonds simultaneously with both materials. On the contrary, increasing the concentration of S vacancies does not lead to any significant change. Finally, we found a slight increase of *d*_Au–MoS_2__ up to 2.76 Å at 11% defect density, which is the result of the atomic rearrangement of the surface Au atoms of the electrode when in contact with the substitutional Au atoms in MoS_2_.

**Fig. 5:**
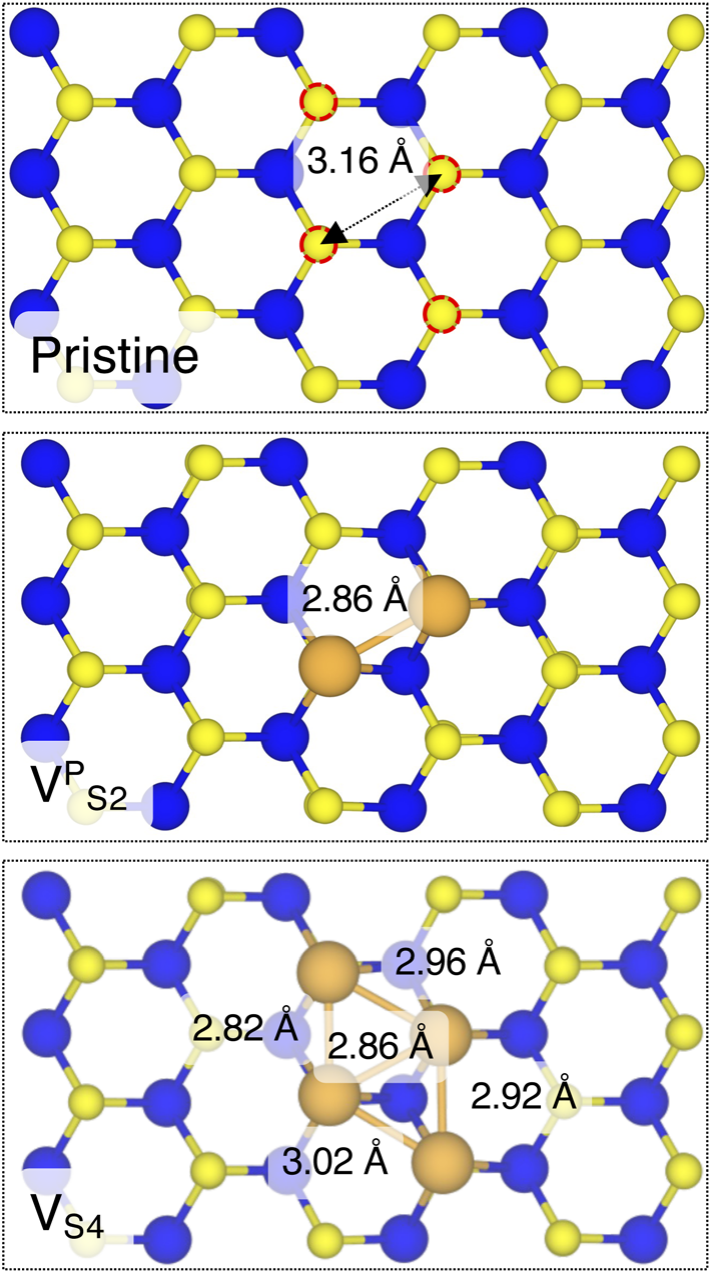
Top view of pristine, V^P^_S2_, and V_S4_ interfaces, where the Au electrode was removed for clarity.

Finally, we considered small and bigger clusters of defects at the Au/MoS_2_ interface. This is relevant, as S vacancies in MoS_2_ have been shown to be rather mobile and thus possibly prone to clustering.^52–54^ Our computed formation energies of defects also corroborate this (see Table S1 in the ESI†). Au atoms migrating from the electrodes may then fill such clusters, forming in turn Au clusters. Here, we focused on S di-vacancy and substitutional Au either in or out of plane MoS_2_. Moreover, we also considered a large 4-atom cluster of S vacancies. We found that both V^A^_S2_ and Au2^A^_S2_ interfaces (*i.e.*, with axial defects) are characterized by the same *E*_ad_ and roughly the same *d*_Au–MoS_2__ of their corresponding V_S_ and Au_S_ interfaces. This is justified by effectively having only one defect in MoS_2_ in contact with the Au electrode, whereas the other is on the opposite side of the material in contact with the vacuum. Therefore, these results suggest that defects on the opposite (bottom) surface of the material have very little or no effect on the structure and the interface energetics of the (top) Au/MoS_2_ contact. In the presence of both top and bottom contacts, each vacancy will then play the same role with the Au electrode in proximity. On the contrary, when we coupled MoS_2_ with in-plane clusters of two and four S vacancies with the metal electrode, we observed the strong tendency of the Au atoms at the bottom layer of the electrode to migrate and fill such clusters, in accordance with previous studies in the literature.^35^ Remarkably, we found that even a small cluster of two S vacancies was enough to cause the Au electrode to partially collapse on MoS_2_. We point out that this was not the case when we randomly introduced the same number of S vacancies per unit cell without any clustering, meaning that their relative position can dramatically change the interaction between materials at their interface. Interestingly, when looking at the structures of both V^P^_S2_ and V_S4_ we observed the tendency of the collapsed Au atoms to reorganize and form small metal clusters, rather than just sit in the position of S vacancies. Indeed, from Fig. 5 one can observe that the Au–Au bond lengths are in every case smaller than the typical distance of 3.16 Å of S vacancies. As expected, the computed *E*_ad_ of both interfaces are very high, confirming the strong interaction between MoS_2_ and the collapsed Au electrode. The final interface considered in this work was Au2^P^_S2_, which essentially corresponds to the situation in which two Au atoms adsorbed on the metal electrode migrate to fill a small cluster of two S vacancies (or two substitutional Au atoms migrate on the MoS_2_ surface to eventually coalesce). For this interface, we found the same values of both *d*_Au–MoS_2__ and *E*_ad_ as in 2 × Au_S_, suggesting that when no collapse of the electrode occurs the relative position of substitutional Au atoms has little influence on the quality of the contact.

### 3.2 Tunnelling probabilities of defective interfaces

**Fig. 6:**
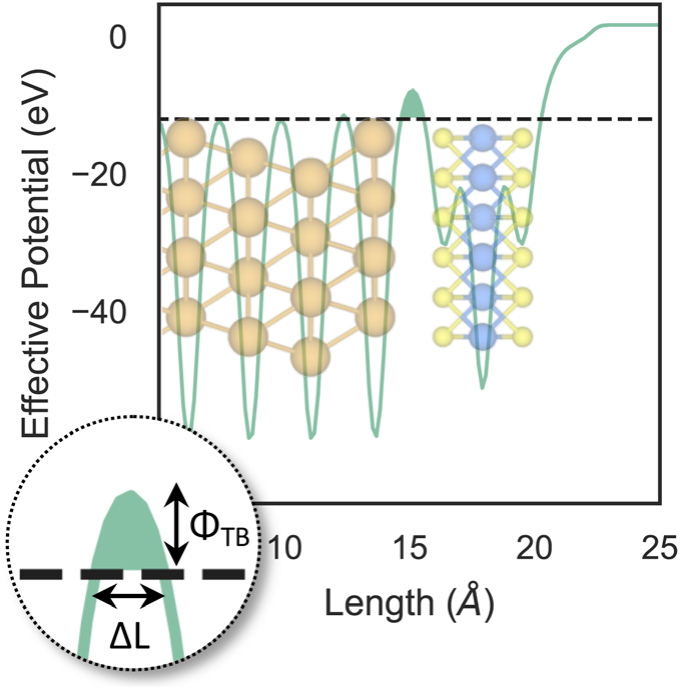
Effective potential across the interface of the pristine Au/MoS_2_ structure. The potential was projected in the direction of the interface. Highlighted in the figure are the tunnelling barrier height (*Φ*_TB_) and the barrier width (Δ*L*).

So far, we have only looked at the structural properties of interfaces. In this part of the work, for each interface considered in this study, we computed electron tunnelling barriers and probabilities as an estimate of the charge transport across the Au/MoS_2_ contact. In device contacts where no chemical bonding occurs between the metal and the semiconductor (as in the structures here considered), a tunnelling barrier is always present.^50,55^ Its magnitude correlates with the contact resistance: a high tunnelling barrier implies a low probability of electrons to cross the metal–semiconductor interface, and thus an expected high contact resistance. To extract the tunnelling probability, we computed the plane averaged effective potential (*V*_eff_) across the interface of every Au/MoS_2_ contact considered in this work, and we projected it in the direction of the interface between the two materials. This quantity is shown in Fig. 6, which refers to the defect-free situation. The barrier height (*Φ*_TB_) is essentially the difference between *V*_eff_ in the Au electrode and at the interface, as highlighted in the figure. Δ*L* is instead the barrier width. Although both *Φ*_TB_ and Δ*L* can provide a first estimate of how intimate (or poor) the metal–semiconductor contact is, the tunnelling probability (*T*) provides a better quantitative figure of merit. We computed *T* by integrating *V*_eff_ between the limits defined by the barrier width (see Fig. 6):2
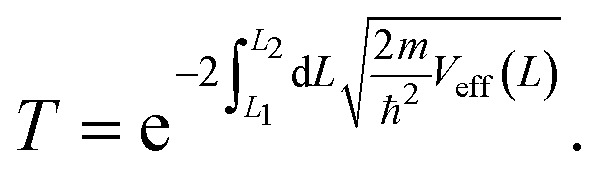


In the above equation, *m* and ℏ are the electron effective mass and the reduced Planck's constant, respectively.

The computed barrier widths, heights, and the tunnelling probabilities are summarized in Table 2. As we found in our previous work,^39^ the tunnelling probability in the defect-free interface is close to 40%. This quantity slightly decreases to 38–39% when introducing S vacancies, suggesting the worsening of the contact quality regardless of the defect concentration. Thus, with a lower value of *T* such defective interfaces are expected to have an even larger contact resistance compared to the pristine interface. On the other hand, Au substitution was found to increase the tunnelling probability of the interface from 44.9% at the lowest concentration (Au_S_) up to 71.1% at the highest concentration (4 × Au_S_). Interestingly, *T* correlates very well with the Au concentration, whereas we observed no correlation when increasing the concentration of S vacancies in MoS_2_, at least until the maximum defect density here considered (11%). This suggests that S vacancies do not play any significant active role in the coupling between MoS_2_ and the electrode. On the contrary, the effect of Au concentration in the tunnelling probability is evident, suggesting a constant improvement of the electron injection rate as more conductive “bridges” are generated at the interface. It is also worth noting that the value of *T* in 2 × Au_S_ and Au2^P^_S2_ is comparable (50.5 and 50.9%, respectively), whereas in V^P^_S2_ (*i.e.*, when the electrode collapses) *T* is 54%. These results suggest that, at equal concentrations of substitutional Au atoms: (i) when the extra Au atoms derive from the Au electrode as adsorbed adatoms, the effect of clustering on the electron injection rate is negligible; (ii) when the extra Au atoms derive from the collapsed electrode, the tunnelling probability is higher. This can also be observed when comparing values of *T* in 4 × Au_S_ against V_S4_ (*i.e.*, when the electron collapses): 71.1 and 89.6%, respectively. We believe this effect to be due to the significant atomic distortion of the Au electrode upon its collapse on MoS_2_, which effectively leads to a shorter average interface distance than that observed when randomly distributing substitutional Au atoms at the materials' interface.

**Table 2:** Computed barrier width (Δ*L*), barrier height (*Φ*_TB_), and tunnelling probability (*T*) of all the Au/MoS_2_ interfaces considered in this work

Interface	Δ*L* (Å)	*Φ*_TB_ (eV)	*T* (%)
Pristine^39^	0.851	3.54	39.7
V_S_^39^	0.875	3.55	38.5
Au_S_	0.773	2.94	44.9
2 × V_S_^39^	0.891	3.60	38.3
4 × V_S_^39^	0.891	3.58	38.2
2 × Au_S_	0.641	2.40	50.5
4 × Au_S_	0.333	0.85	71.1
V^A^_S2_ (ref. 39)	0.868	3.64	39.0
V^P^_S2_	0.560	1.97	54.0
V_S4_	0.110	0.23	89.6
Au2^A^_S2_	0.729	2.90	45.0
Au2^P^_S2_	0.627	2.33	50.9

### 3.3 Electronic structure of MoS_2_ in defective interfaces

**Fig. 7:**
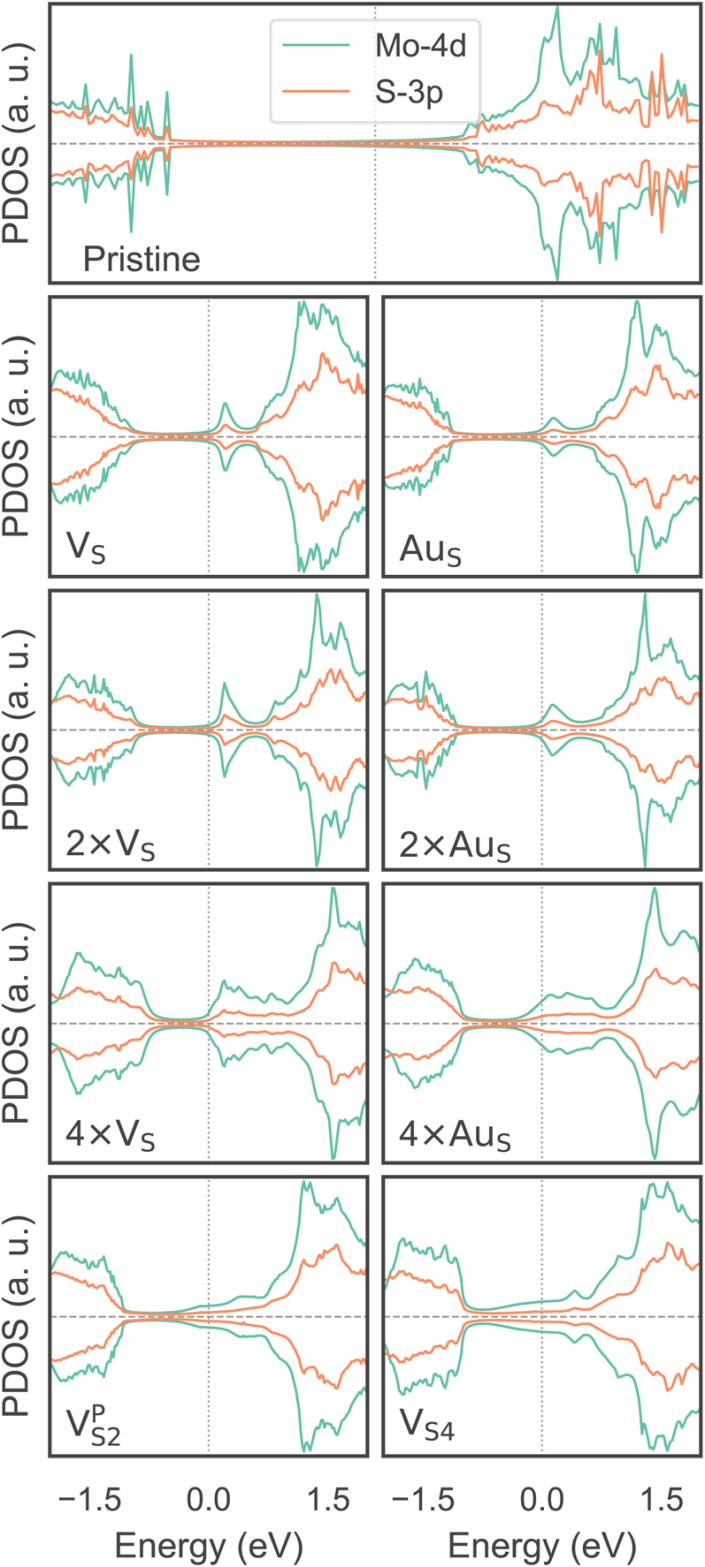
Density of states of selected interfaces, projected onto the Mo-4d and S-3p orbitals of MoS_2_. The plots, which result from the Green's function surface simulations, are centered around the Fermi level of each system (vertical dotted line).

To investigate how the electronic properties of MoS_2_ within the contact change due to the presence of defects, we computed the density of states (DOS) of selected interfaces, projected onto the Mo-4d and S-3p orbitals of MoS_2_. The projected DOS (PDOS) plots are shown in Fig. 7.

As expected, the pristine MoS_2_ retains its band gap also in the presence of the Au electrode. We computed its value to be ∼1.75 eV, in excellent agreement with experimental data.^20^ We note, however, the shift of the Fermi level towards the conduction band, indicating the n-type doping effect of the Au electrode. In general, the presence of S vacancies does not change the current picture: the material retains its semiconducting properties, although we observed the formation of intra-gap states in the proximity of the conduction band. At a defect density of 11% (4 × V_S_), several intra-gap states merging with the conduction band of MoS_2_ are observed. Nevertheless, the Fermi level of the system is still found within the material's band gap, although being very close to the new conduction band edge.

The presence of substitutional Au atoms also leads to intra-gap states close to the conduction band edge of MoS_2_. Interestingly, at the high defect density of 11% (4 × Au_S_) we found such intra-gap states to cross the Fermi level of MoS_2_, suggesting the metallization of the material. We point out that this is consistent with our previous results on adhesion energies and tunnelling probabilities, which both pointed towards the enhanced interaction between the electrode and MoS_2_, and thus to an intimate device contact. We observed the metallization of MoS_2_ also in both V^P^_S2_ and V_S4_, where our simulations showed the collapse of the Au electrode. Notably, the PDOS of V_S4_ shows the complete vanishing of the material's band gap, which indeed correlates with the highest value of *T* (89.6%) computed in this work, among all the interfaces considered.

Overall, our results show that substitutional Au atoms at higher concentrations cause MoS_2_ to switch from a semiconductor to a metal, and this is expected to significantly change the resistance at the device contact (see Table 2). Also, we could confirm that the conduction in the LRS of the atomristor is indeed metallic, as it was previously argued by experimental studies.^22,30^

### 3.4 Mechanism of the resistive switching

**Fig. 8:**
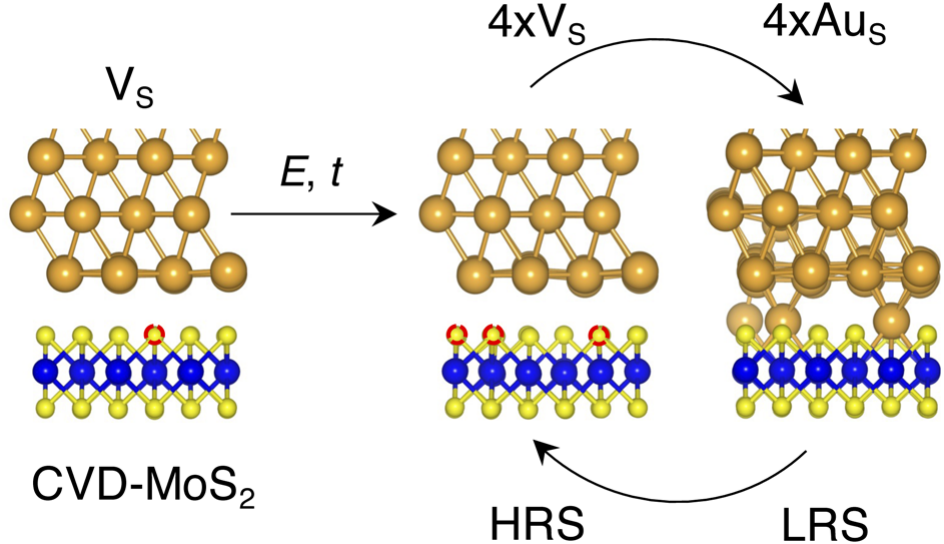
Illustration of the proposed resistive switching mechanism and the possible Au/MoS_2_ interfaces involved. Here, we propose the electric field (*E*) and/or temperature (*t*) as key quantities to generate extra S vacancies and to force the migration of Au atoms from (to) the metal electrode.

Based on the results presented so far, we propose the following steps behind the resistive switching in vertical Au/MoS_2_/Au memristors:

1. CVD-MoS_2_ with its typical S vacancy density (∼10^13^ cm^−2^) is in contact with the Au electrode. This is well represented by the interface V_S_, as shown in Fig. 8.

2. As the device is switched on for the first time, extra S vacancies are generated due to either the presence of the electric field or the increase in the local temperature. This situation corresponds to the HRS. Among the interface structures considered in this study, 4 × V_S_ (*T* = 38.2%) would correspond to the best case scenario in which the highest density of S vacancies is obtained (see Fig. 8).

3. Extra Au atoms migrate from the electrode to MoS_2_, where they are able to fill the S vacancies. The Au migration may be due to both the electric field and the Joule heating caused by it. The computed charge distribution of selected interfaces (see Fig. S1 in the ESI†) shows that extra Au atoms assume a positive partial charge, thus confirming the susceptibility of such atoms to the electric field. The substitutional Au atoms in MoS_2_ act as conductive “bridges”, thus allowing electrons to easily flow across the contact. In this situation, MoS_2_ becomes metallic and this corresponds to the LRS. Assuming the highest density of S vacancies, the LRS is best represented by the interface 4 × Au_S_ (*T* = 71.1%), as can be seen in Fig. 8.

4. To reset the device, an opposite electric field may be applied, forcing substitutional Au atoms to migrate back to the metal electrode, thus restoring the HRS.

In this picture, we note that extra Au atoms may derive either from adatoms adsorbed on the Au(111) surface or directly from the electrode upon its collapse on MoS_2_, provided a (small or large) cluster of S vacancies is present on the material's surface. As previously discussed, clusters of vacancies may form due to the mobility of S vacancies in MoS_2_. This process is usually aided by the presence of the electric field or the increase in the local temperature due to self-heating in the device. Assuming the collapse of the Au electrode, resetting the device may not always be possible: very high temperatures and a high electric field may both be needed. This suggests that the electrode collapse could lead to a failure mechanism in which the device is stuck in the LRS. The formation of clusters of S vacancies may then be detrimental.

## 4 Conclusions

To conclude, by means of a computational modelling approach which combines DFT and Green's function surface simulations, we unravelled the physics of the resistive switching in vertical memristors based on single-layer MoS_2_ and Au electrodes. To achieve this, we carried out several *ab initio* simulations of pristine and defective Au/MoS_2_ interfaces with either S vacancies or substitutional Au atoms. Also, we varied the concentration of such defects and we considered the formation of defect clusters. To shed light onto the memristive mechanism, we explored the impact of defects on the structural and electronic properties of interfaces, and on their tunnelling probabilities.

Our simulations revealed that the switch between HRS and LRS in MoS_2_-based memristors is not due to the mere presence of S vacancies in CVD-MoS_2_, but rather to the migration of Au atoms from the metal electrode to MoS_2_. When migrating, these extra Au atoms occupy the position of the S vacancies and are able to act not only as physical but also as conductive “bridges” between the two materials, enhancing the transfer of electrons from the electrode to MoS_2_. Based on such findings, we believe that the HRS corresponds to the situation in which only S vacancies are present in the material, whereas the LRS is achieved when a sufficient number of Au atoms have migrated and filled the S vacancies in MoS_2_, which then becomes metallic. This hypothesis is corroborated by the interface geometries, clearly showing the extra Au atoms to simultaneously bind with MoS_2_ and the Au electrode. Also, the computed tunnelling probabilities of electrons are significantly higher only in the presence of substitutional Au atoms in MoS_2_. On the contrary, when only S vacancies are present in the material the tunnelling probability is low and comparable to that of the defect-free interface. The PDOS plots of the interfaces confirm this, and show the metallization of MoS_2_ at higher concentrations of substitutional Au atoms. We also found that extra Au atoms may derive either from adatoms adsorbed on the Au surface, or directly from the electrode upon its collapse on MoS_2_. The electrode collapse was observed only if clusters of S vacancies are present in the material and could lead to a failure mechanism in which the device is stuck in the LRS.

We point out that the results presented in this work not only support the experimental findings in the literature, but also provide a theoretical understanding of the resistive switching mechanism in vertical Au/MoS_2_/Au memristors. We hope our work will contribute towards the optimal design and development of 2D memristors.

## Author contributions

Gabriele Boschetto: conceptualization, methodology, validation, formal analysis, investigation, data curation, writing – original draft, writing – review & editing, visualization; Stefania Carapezzi: conceptualization, writing – review & editing; Aida Todri-Sanial: conceptualization, writing – review & editing, supervision, project administration, funding acquisition.

## Conflicts of interest

There are no conflicts to declare.

## Supplementary Material

NA-005-D3NA00045A-s001
