# Supplementary material for: Non-volatile resistive switching mechanism in single-layer MoS2 memristors: insights from ab initio modelling of Au and MoS2 interfaces
Source: Nanoscale Adv. 2023 Jul 21;5(16):4203–12. doi: 10.1039/d3na00045a (PMC10408618; doi:10.1039/d3na00045a)
Supplement: NA-005-D3NA00045A-s001 [file NA-005-D3NA00045A-s001.pdf]

Electronic Supplementary Material (ESI) for Nanoscale Advances.
This journal is © The Royal Society of Chemistry 2023

Supporting Information

Non-volatile resistive switching mechanism in single-layer MoS_2 memristors: Insights from ab initio modelling of Au and MoS_2 interfaces

Gabriele Boschetto,[1] Stefania Carapezzi[1], and Aida Todri-Sanial[1,2]

[1]Laboratory of Computer Science, Robotics, and Microelectronics, University of Montpellier, CNRS, 161 Rue Ada, 34095 Montpellier, France
[2]Department of Electrical Engineering, Eindhoven University of Technology, Groene Loper 3, 5612 AE Eindhoven, Netherlands

E-mail: gabriele.boschetto@lirmm.fr; aida.todri@lirmm.fr

1. Stability of defects in free-standing MoS_2

We assessed the effect of S vacancies, Au substitution, and different combinations of both on free-standing monolayer MoS_2 by computing formation energies of selected defects.

Table S1. *Formation energies of selected defects in free-standing MoS_2.*

Defect	$E_F[MoS_2]$ (eV)	$E_F[V_S]$ (eV)
V_S	2.75	-
V_{S2}^A	5.45	2.69
V_{S2}^P	5.48	2.73
Au_S	3.48	0.72
$Au2_{S2}^A$	7.27	4.52
$Au2_{S2}^P$	6.64	3.88

The structural stability of defects can be assessed by computing their formation energies (see Table S1). To compute such a quantity, we considered two cases: i) a defect-free MoS_2, and ii) a MoS_2 sample with a V_S density of ~2%, in line with typical defect densities found in CVD-MoS_2. Formation energies computed with the latter approach ($E_F[V_S]$) give us an estimate of how likely it is to generate new S vacancies (and Au to fill such vacancies) when the material is already defective, as it is the case in MoS_2 nanodevices. Formation energies computed with defect-free MoS_2 ($E_F[MoS_2]$) confirmed that V_S is the most thermodynamically stable defect ($E_F[MoS_2]$ = 2.75 eV), in accordance with previous studies. Additionally, it is worth noting that $E_F[MoS_2]$ of an axial (V_{S2}^A) and an in-plane S di-vacancy (V_{S2}^P) is lower than the sum of $E_F[MoS_2]$ for two infinitely separated S vacancies, which suggests the slight tendency of V_S to form clusters. We also found that Au_S has a higher $E_F[MoS_2]$ (3.48 eV) than a simple S vacancy, which is the outcome of both the removal of an S atom and the addition of an Au atom from/to the defect-free MoS_2. We could also observe the tendency of Au atoms to cluster in-plane rather than axially. When considering the material with a V_S density of ~2%, we were able to confirm that additional S vacancies are slightly more likely to form in the proximity of

an already existing vacancy. Indeed, $E_F[V_S]$ for one additional S vacancy is slightly lower than $E_F[MoS_2]$ for the single S vacancy. Moreover, we found that the formation energy of Au_S significantly decreases ($E_F[V_S]$ = 0.72 eV), making this the most energetically favourable defect. Once again, we found that Au atoms tend form small clusters in-plane rather than axially out-of-plane.

2. Partial Charges of Selected Interfaces

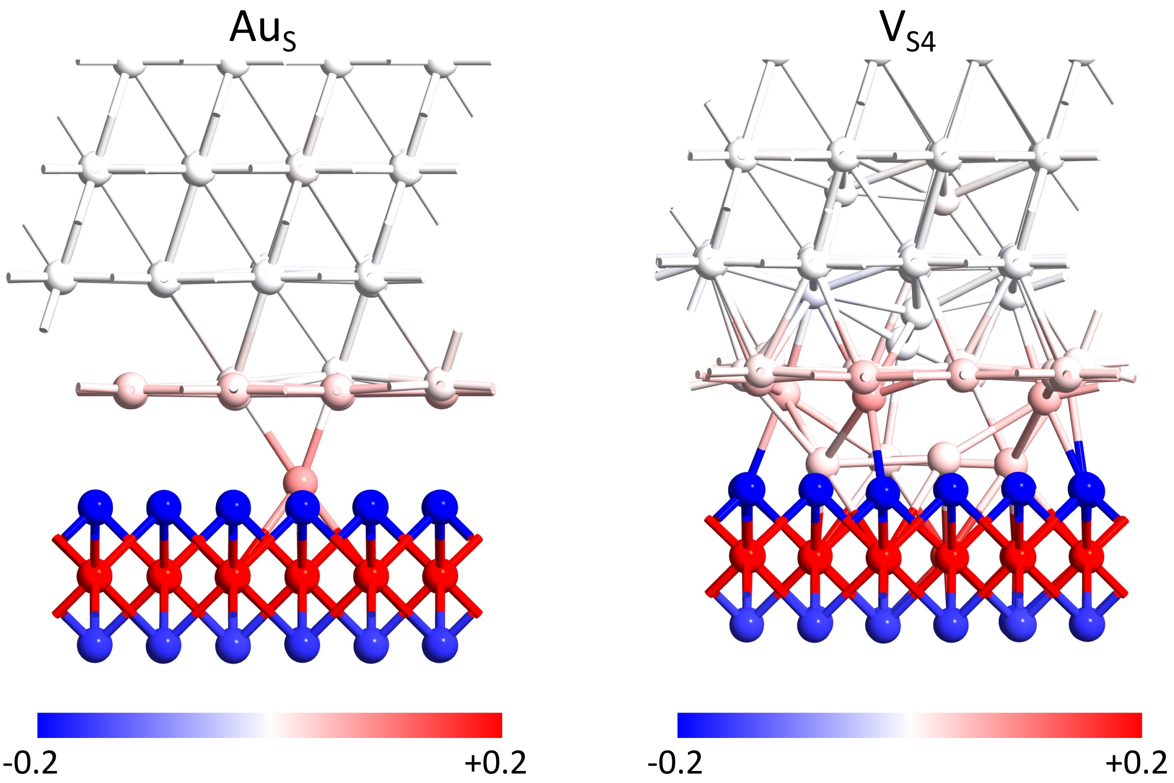

Figure S1. *Mulliken population analysis of Au_S and V_{S4} interfaces. Negative partial charges are in blue, whereas positive partial charges in red.*
